# Knocking out consumer concerns and regulator’s rules: efficient use of CRISPR/Cas ribonucleoprotein complexes for genome editing in cereals

**DOI:** 10.1186/s13059-017-1179-1

**Published:** 2017-02-28

**Authors:** Felix Wolter, Holger Puchta

**Affiliations:** Botanical Institute, Karlsruhe Institute of Technology, Fritz-Haber-Weg 4, Karlsruhe, 76131 Germany

## Abstract

Selection-free genome editing using Cas9 ribonucleoprotein embryo bombardment has been achieved for maize and wheat. This is a breakthrough that should make new breeding technologies more acceptable for worldwide use.

## Introduction

Two recent publications show that it is possible to use CRISPR/Cas ribonucleoproteins (RNPs) to achieve selection-free site-directed mutagenesis by bombarding embryos of the main crop plants maize [1] and wheat [2]. But why is this exciting given that CRISPR/Cas technology has been transforming plant biology for years? Multiple new tools have been developed for plant genome engineering [3] and it has become possible to edit a greater variety of plant species [4]. Application of the technology is becoming more attractive for agronomical purposes. Recently, a number of genome-edited crops with attractive traits have been produced [5]. The use of CRISPR/Cas RNPs for mutation induction, first shown in human cells [6], has been achieved in protoplasts of several plant species [7]. Two recent publications in *Nature Communications* from Svitashev et al. [1] and Liang et al. [2] demonstrate that the use of RNP-mediated editing is now possible for two of the world’s most important crop plants, and that the farming of the resulting plants with improved traits should not be blocked by regulation hurdles worldwide as they cannot be regarded as genetically modified organisms (GMOs).

## Different standards worldwide: is the process or product relevant?

When coming to the question of when a crop should be regarded as GMO, in many cases the answer you get will depend on the place where you ask it, at least from a legal point of view. From the scientific point of view, the answer is easy: if the respective plants cannot be discriminated from a natural variant, which will always apply for plants that carry an induced mutation of one or a few changed nucleotides without a transgene insertion in their genomes, it is completely pointless to classify them as GMOs. One always has to keep in mind that classic mutagenesis by chemicals, as well as radiation, is widely used for the production of new varieties of crops.

Radiation-induced double-strand breaks (DSBs) are also repaired by the same “natural” pathways as CRISPR/Cas [5]. The drawback of the classic strategy is that attractive mutations can only be obtained in an undirected manner and at the cost of many more unwanted changes in the same genome that can only partly be eliminated from the final product by outcrossing. Nevertheless, over 3000 crop varieties have been produced over the years using radiation mutagenesis and are used worldwide without the slightest legal restriction.

In the USA, three agencies are responsible for the regulation of GMOs: the Department of Agriculture (USDA), the Food and Drug Administration (FDA) and the Environmental Protection Agency (EPA). The evaluation mainly takes into account the end product of the procedure that is planted in the field by the farmer. Thus, only plants with transgenes permanently integrated into the genome are regarded as GMOs. The current US regulation has already classified a number of crops mutated by synthetic nucleases as non-GMOs, as they contain an induced mutation but no transgene [5]. In contrast, in Europe, EU legislation defines GM crops specifically as “an organism (…) in which the genetic material has been altered in a way that does not occur naturally by mating and/or natural recombination”. Here, the process is also relevant, as GMO-critical organizations argue that any recombinant nucleic acid, even if only transiently applied or outcrossed from the product before planting, defines the respective plant as GMO even in the absence of the transgene in the end product. This interpretation is fortunately not generally accepted within the member states. Interestingly, at the end of 2015, the Swedish Board of Agriculture (Sweden is part of the EU) confirmed the interpretation that some plants which have had their genomes edited using CRISPR/Cas9 technology do not fall under the European GMO definition. Nevertheless, due to public concerns, a restrictive interpretation of process principle might become an important hurdle in the future for the use of gene-edited plants within the EU, although such a regulation would never be experimentally verifiable. As the decision of the EU might be a guiding light for a number of other nations, such a decision would have worldwide consequences.

To avoid such risks, the idea arose to modify genome-editing approaches in such a way that the synthetic nuclease is not expressed in the transformed cell from a recombined nucleic acid, but is delivered in its active form similar to chemical mutagens in classic breeding. Therefore, plants where mutations are induced by proteins such as synthetic nucleases or RNPs, for example Cas9, should not fall under the current EU regulation and qualify the respective plants as non-GMOs.

## DNA-free genome editing in wheat and maize established

Due to the presence of the cell wall, efficient delivery of genome-editing reagents into intact plant cells is mainly limited to two methods: *Agrobacterium*-mediated delivery of transfer DNA (T-DNA) and biolistic delivery of plasmid DNA. In both cases, the delivered DNA frequently integrates into the plant genome. Now, two recent pioneering studies have demonstrated the potential of the idea to edit plant genomes without introducing foreign DNA. The group of Caixia Gao from the Chinese Academy of Science in Beijing had already shown that it is possible to achieve a high rate of edited plants without transgene integration if the use of selectable markers is omitted [8]. In this approach, they delivered Cas9 and sgRNA on DNA vectors without selectable markers into immature wheat embryos via particle bombardment. Plants were regenerated without selective agents within 6–8 weeks, which is a significantly shorter time period than earlier protocols using selective agents [9]. Mutant analysis revealed that more than half of the regenerated mutant plants contained no transgene. They further demonstrated that Cas9 and sgRNA can be transcribed in vitro and delivered in the form of RNA. Since RNA cannot integrate into the genome, the obtained mutants are transgene-free, but mutagenesis efficiency was lower when RNA was delivered.

**Fig. 1:**
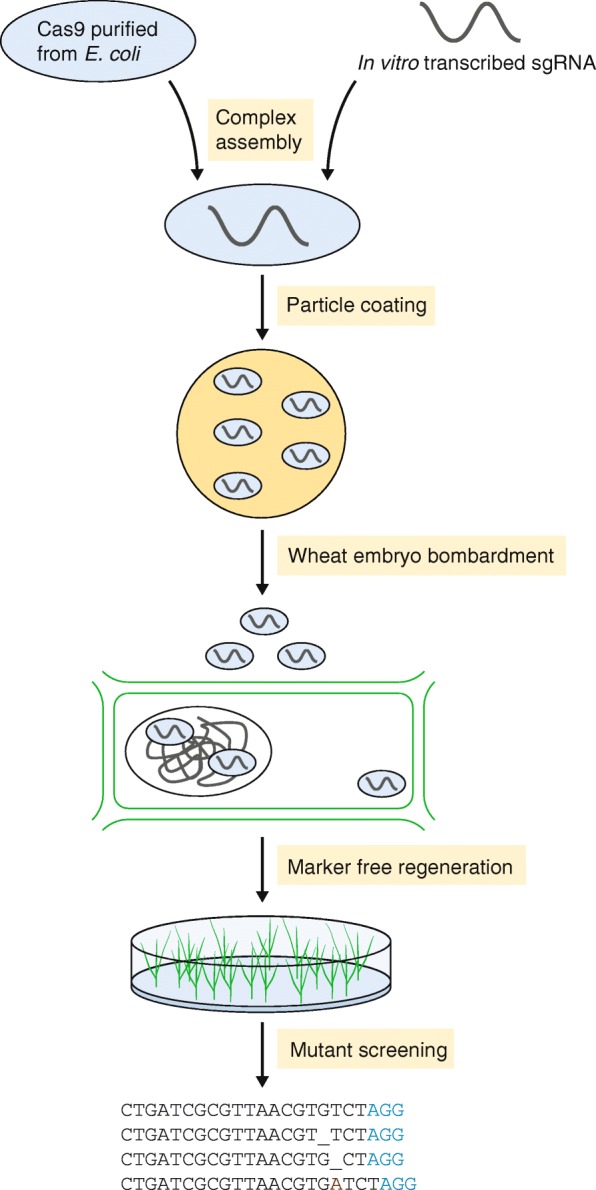
Workflow used by Liang et al. [2] to achieve DNA-free editing of wheat genes. Cas9 is expressed in *E. coli* and purified. Single guide RNA (*sgRNA*) is transcribed in vitro and complexed with Cas9. This complex is coated onto 0.6 μm gold particles which are then bombarded into immature wheat embryo cells. Plants are regenerated without any selective agent from bombarded embryos and screened for mutations via PCR/restriction enzyme assay and sequencing

More recently, the same group expressed Cas9 in *Escherichia coli* and pre-assembled it with in vitro transcribed guide RNAs targeting two different wheat genes [2]. After functional validation of these RNPs in protoplasts, they were delivered into immature embryo cells of wheat via particle bombardment. Again, plantlets were regenerated from bombarded embryos without the use of any selective agents within 6–8 weeks (Fig. 1). While on-target mutagenesis of RNP delivery (up to 4.4% of regenerated plantlets showed target mutations) was comparable to DNA delivery, mutagenesis at an off-target site harbouring a single nucleotide mismatch was considerably reduced. In contrast, conventional DNA delivery led to mutagenesis at the off-target site that was comparable to on-target mutagenesis.

The group of Mark Cigan from DuPont Pioneer, Johnston USA, performed similar experiments in maize [1]. They delivered pre-assembled RNPs targeting four different genes into immature embryo cells via particle bombardment. Again, plants were regenerated without selectable markers and, depending on the target, 2.4–9.7% of plants showed mutated alleles. Notably, not a single regenerated plant showed mutations at an off-target site harbouring two PAM distal mismatches. Just as in wheat, off-target mutations were only detectable by amplicon deep sequencing of bombarded embryos. In maize, RNP delivery also enabled homologous recombination (HR)-mediated precise gene editing of the endogenous *ALS2* when a 127-bp single-stranded repair template for HR was co-delivered. This demonstrates the much broader applicability of RNP delivery than mere gene disruption.

In addition to the discussed benefits concerning legal regulation, this new technology has two additional advantages. First, off-site effects were drastically reduced compared with conventional DNA delivery, which is clearly beneficial for the development of new crop varieties. Second, when DNA is integrated into the genome in the breeding process it needs to be segregated away by backcrossing. However, this can be quite time consuming in the case of crops such as wheat and maize with complex genomes and/or long breeding cycles. This additional effort can be safely omitted with this new exciting technology.

## Closing remarks

We will only be able to address challenges of the world, including ensuring sufficient food supply, if we evaluate newly developed technologies for their risk potential and their sustainability in a rational way. Hopefully, the two publications discussed here will help to achieve this goal and make the CRISPR/Cas technology more accessible for use in agriculture all over the globe.

## Abbreviations

GMO: Genetically modified organism
RNP: Ribonucleoprotein
sgRNA: Single guide RNA
